# Effects of glucosyl-hesperidin and physical training on body weight, plasma lipids, oxidative status and vascular reactivity of rats fed with high-fat diet

**DOI:** 10.2147/DMSO.S153661

**Published:** 2018-07-04

**Authors:** Tiago Tomazini Gonçalves, Carolina M Lazaro, Fernanda G De Mateo, Marcela CB Campos, Jackeline GB Mezencio, Mario A Claudino, Patrícia de O Carvalho, R Clinton Webb, Fernanda BM Priviero

**Affiliations:** 1 Laboratory of Multidisciplinary Research, Universidade São Francisco, Bragança Paulista, São Paulo, Brazil, fernanda.bmp@gmail.com; 2 Department of Physiology, Medical College of Georgia, Augusta University, Augusta, GA, USA, fernanda.bmp@gmail.com

**Keywords:** high-fat diet, physical activity, oxidative stress, antioxidants, obesity, physical exercise, supplementation, flavonoids, reactive oxygen species

## Abstract

**Objective:**

The aim of the study was to evaluate the effects of supplementation with glucosyl hesperidin (GH), with or without physical training, on body weight, fat depot, glucose and plasma lipids, oxidative status and vascular function of rats fed with high-fat diet (HFD).

**Methods:**

After weaning, male Wistar rats were fed with an HFD plus fructose for 12 weeks and started receiving oral antioxidant supplementation and/or physical training after the fourth week of diet for eight further weeks. Body weight, epididymal and retroperitoneal fat, plasma glucose and lipids, oxidative status and mesenteric artery reactivity were evaluated.

**Results:**

Rats fed with HFD presented higher body weight gain and fat accumulation compared to control rats, while GH supplementation did not influence these parameters. Physical training reduced the body weight gain and fat accumulation and modulated the oxidative status by increasing superoxide dismutase activity and total antioxidant capacity and reducing lipid peroxidation. GH alone decreased lipid peroxidation. However, when given to exercised rats, it impaired the response elicited by physical training. HFD caused endothelial dysfunction, and neither GH nor physical exercise prevented it. Potency of sodium nitroprusside was increased in exercised animals but not in GH-supplemented rats.

**Conclusion:**

Physical exercise partially decreased the body fat accumulation, decreased plasma levels of glucose and lipids and improved general oxidative status and endothelium-independent relaxation in mesenteric arteries of rats fed with HFD. GH exhibited benefits only in the oxidative status. However, GH given in association with physical exercise did not cause further changes in addition to those promoted by physical exercise. On the contrary, in exercised animals, GH prevented those changes elicited by physical training in plasma glucose and lipids, oxidative status and endothelium-independent relaxation.

## Introduction

Obesity, which is characterized by excessive fat accumulation, is a multifactorial etiology of chronic disease, and excess adiposity may be related to genetic predisposition, environmental factors, lifestyle and also emotional factors.^1^ Previously, adipose tissue was considered only as a simple reservoir of energy. More recently, adipose tissue has been seen as a complex organ with several functions, contributing to the endocrine system and releasing proinflammatory adipokines which influence regulatory processes. Thus, diseases such as diabetes, atherosclerosis, hypertension, dyslipidemias and coronary artery disease may have adipose tissue as a contributing factor for their pathophysiology.^2^

It is well known that the free radicals and reactive oxygen species (ROS) are products of the cellular metabolism and have physiological functions such as synthesis of cellular structures and play an important role in activation of the immune system. However, the imbalance between production and capability of clearing ROS leads to oxidative stress, which harmfully affects cellular structures such as membranes, lipids, proteins, lipoproteins and DNA.^3^ Obesity is related to oxidative stress and increases lipid peroxidation through three main mechanisms.^4^ Obese individuals have higher oxygen consumption because of increased metabolic rates of the myocardium, which enhances the production of ROS due to enhanced mitochondrial respiration and, therefore, may cause lipid peroxidation.^5^ In addition, the excess of body mass might cause a cumulative and progressive pressure, leading to cell injury with secretion of cytokines such as tumor necrosis factor (TNF), which generates ROS and, thus, increases lipid peroxidation.^6^ In addition, a recent study in human beings revealed a positive correlation between body mass index and malondialdehyde (MDA) – one product of lipid peroxidation. The level of MDA is also inversely related to superoxide dismutase (SOD), which indicates that in obesity, depletion of antioxidant enzymes may occur. It was also observed that the more serious obesity is, the more lipid peroxidation and less protective enzymes are found.^7^ Furthermore, it is well described in the literature that the imbalance in the redox state can damage cells and may lead to vascular dysfunction, atherosclerosis, hypertension and several other secondary diseases.^8^ Finally, it is possible that a diet rich in fatty acids changes oxygen metabolism since fatty acids containing double bonds are molecules more vulnerable to oxidative reactions, which can lead to lipid peroxidation.^3^

The antioxidant system is divided into two mechanisms: enzymatic and nonenzymatic antioxidants. The primary enzymatic defense consists of three main endogenous enzymes, such as glutathione peroxidase (GPx), SOD and catalase (CAT), which act to prevent the formation of free radicals or neutralize their action. Precisely, GPx donates electrons to reduce peroxidase, and CAT converts hydrogen peroxide (H_2_O_2_) into water and oxygen molecule, while SOD converts superoxide anions (O_2_^−^) into hydrogen peroxide as a substrate for CAT.^9^ Nonenzymatic defense system comprises antioxidant compounds of dietary origin, emphasizing vitamins, minerals and phenolic compounds.^10^ Hesperidin is a citric flavonoid, considered as a glycosidic flavanone, and its chemical structure is constituted by hesperetin (aglycone form) and linked to glucose sugars and rhamnose in position 7.^11^ It is naturally present in citrus fruits, especially in orange juice, and their amounts may vary according to soil, climate, fruit variety and type of juice.^12^ It has been described that flavonoids have lipid-lowering, anti-inflammatory, antitumor, antioxidant and antiviral properties.^13^ Despite its benefits, hesperidin has low solubility in water (<0.01%), reducing its bioavailability to the body. Therefore, a synthetic form of hesperidin called glucosyl hesperidin (GH), which is 10,000 times more soluble than hesperidin, was developed through the transglycosylation with cyclodextrin glucanotransferase from the bacterium *Bacillus stearothermophilus*. Biological activities of GH were shown to be equal to or better than those of hesperidin. In addition, in rats, absorption and urinary excretion of GH compared to hesperidin showed the same profile of metabolites excreted, while GH was more efficiently absorbed due to high solubility.^11^ In rats, we have previously compared the bioavailability of hesperidin, GH and hesperetin. It was observed that hesperetin was the major form of flavonoid present in plasma, although GH had similar bioavailability, which suggests that GH might exhibit therapeutic potential.^14^

Beyond dietary antioxidant products, physical exercise training has been considered a stimulus for the endogenous antioxidant system. It is believed that physical activity enhances the production of ROS, and therefore, it increases the synthesis of endogenous antioxidant enzymes to combat oxidative stress. In fact, a previous study showed that exercise increases NF-κB to promote gene expression of antioxidant enzymes; however, this effect was blocked by the use of antioxidant allopurinol.^15^ Accordingly, it was previously demonstrated that, in healthy subjects, the administration of antioxidants such as vitamin C, vitamin E and α-lipoic acid blunted the health-promoting effects of physical training.^16^–^18^ In this study, we hypothesized that treatment with the antioxidant GH might be beneficial to prevent oxidative stress in obesity. On the other hand, we hypothesized that when administered to animals subjected to physical training, the exogenous antioxidant might interfere with ROS produced by physical exercise and thus decrease the benefits of physical training to augment the endogenous antioxidant defense. We aimed to evaluate the impact of physical training with or without the antioxidant flavonoid, GH, on body weight and fat accumulation, plasma glucose and lipids, oxidative status and vascular reactivity of obese rats.

## Methods

Experimental procedures were carried out according to the general ethical guidelines for animal use established by the Brazilian Society of Laboratory Animal Science [Sociedade Brasileira de Ciência em Animais de Laboratório] and EC Directive 86/609/EEC for animal experiments. The experimental protocols were approved by the ethics and research committee of the Universidade São Francisco (004.06.11 and 001.09.2016).

### Animals

Four-week-old Wistar male rats from Cemib-Unicamp (Campinas, Brazil) were housed in collective cages (five rats per cage) and kept in a light/dark cycle (12/12 h) at a temperature of 22°C ± 3°C, receiving water and chow ad libitum. It is important to note that, to reduce variables which might be gender-specific characteristics, only male rats were used in this study. Thus, the findings of this study may not be applied to female animals.

### Experimental design

Rats were randomly divided into five groups of five rats as follows: 1) control (fed with standard chow); 2) high-fat diet (HFD; fed with HFD + fructose); 3) HFD supplemented with GH (HFD + GH); 4) HFD and subjected to physical training (HFD + TR); and 5) HFD supplemented with GH and subjected to physical training (HFD + GH + TR).

Animal obesity was induced by the association of an HFD (59%) and water containing 100 mg/mL of fructose (LabSynth, Diadema, Brazil) which provided 0.4 kcal/mL, as previously described.^19^ Fructose was added in the tap water of rats receiving HFD only. The HFD was commercially purchased (Prag Solutions Biosciences, Jaú, Brazil) and provided 5.5 kcal/g (being 59% lipids, 18% proteins and 23% carbohydrates). The control group was fed with standard chow for rodents, which provided 3 kcal/g (being 40% carbohydrates, 3.8% lipids and 26.5% proteins). Both diet compositions are summarized in Table 1. HFD and fructose were given for 12 weeks, beginning after weaning at the fourth week of life. Food and water intake were controlled daily, by the difference of the amount offered and the amount left by the animals after a night cycle, and were divided by the number of animals in each cage to estimate the consumption per rat per day. Total energy intake was calculated as food and water consumption times the amount of calories of standard or HFD plus fructose calories in the HFD groups as follows: control group: g × 3 kcal; HFD + fructose groups: g × 5.5 kcal + mL × 0.4 kcal. After 4 weeks of HFD, groups supplemented with GH received 0.5 mmoL/kg of body mass of GH, diluted in saline and administered by daily oral gavage during 8 weeks.^14^ Volume administered did not exceed 0.5 mL. In physically trained animals, exercise program was performed as previously described.^20^,^21^ Briefly, after 1 week of adaptation, rats were subjected to a running program in a motor-driven treadmill, which lasted 8 weeks and was performed in sessions of 60 min, 5 days a week, at 1.2 km/h of speed. Animals were under supervision of an experienced investigator during each session of training. Animals not adapted to the exercise program or which stopped running anytime during the training protocol were removed from the study.

Body weight was evaluated by measuring in the beginning and at the end of the study. After euthanasia, epididymal and retroperitoneal fat pads were isolated and weighed as an estimate of visceral adipose tissue (VAT) depot.^22^

### Biochemical assays

Serum and plasma samples were collected from anesthetized rats after at least 12 h of fasting. During the fasting period, animals had free access to tap water with no fructose. Briefly, arterial blood was collected in dry or EDTA-containing vial, respectively, for serum and plasma taken from the descending branch of the aorta. Plasma and serum were obtained by centrifugation (1000× *g*, 10 min), and levels of glucose, total cholesterol, triglycerides, albumin, total protein and urea were measured by enzymatic method using specific kits (Labtest, Lagoa Santa, Brazil).

All experiments were based on colorimetric assay and performed according to the manufacturer’s instructions. Plates were read in a microplate spectrophotometer (Epoch; BioTek Instruments Inc., Winooski, VT, USA).

### Functional assessment of mesenteric artery

Cranial mesenteric artery was removed and placed in chilled Krebs–Henseleit buffer with the following composition (mmol/L): NaCl, 130; NaHCO_3_, 14.9; dextrose, 5.5; KCl, 4.7; KH_2_PO_4_, 1.18; MgSO_4_7H_2_O, 1.17 and CaCl_2_2H_2_O, 1.6. Tissues were cleaned from connective and adipose tissues and and the arteries were cut into rings (1 mm length) and mounted in a four-chamber wire myograph (DMT, Copenhagen, Denmark) connected to a PowerLab 8/SP™ data acquisition system (software chart 5.0; ADInstruments, Colorado Springs, CO, USA). The bathing solution was maintained at 37°C and continuously aerated with 95% O_2_ and 5% CO_2_. Tissues were allowed to equilibrate for 45 min under a resting tension of 10 mN. Rings were contracted with phenylephrine (PE; 10 μmol/L), and endothelial function was assessed by applying acetylcholine (ACh; 1 μmol/L). After washing out, cumulative concentration–response curves to ACh (0.001–10 μmol/L) or NO donor sodium nitroprusside (SNP; 0.00001–1 μmol/L) were obtained in PE-contracted rings with intact and denuded endothelium, respectively, to evaluate endothelium-dependent and endothelium-independent relaxation. Relaxation was evaluated only in rings that reached a plateau after PE-induced contraction. Vascular relaxation was expressed as the percentage of PE-induced maximum contraction. Potency is given by the pEC^50^ which represents the concentration of each drug necessary to cause 50% of maximum relaxation, and it was expressed as −log molar (mol/L). ACh, SNP and PE were purchased from Sigma-Aldrich Co. (St Louis, MO, USA). All other reagents used were of analytical grade. Stock solutions were prepared in deionized water and stored in aliquots at −20°C; dilutions were made up immediately before use.

### Oxidative status evaluation

Oxidative status was assessed by measuring plasma activity of antioxidant enzymes SOD and CAT, total antioxidant capacity (TAC) and plasma concentration of MDA, this latter measure being an index of lipid peroxidation. For SOD, CAT activity and TAC, plasma was kept on ice for assaying and samples, standards and all reagent enzymes were prepared and performed as described in commercial kit (SOD Assay Kit, CAT Assay Kit and Antioxidant Assay Kit; Cayman Chemical Co., Ann Arbor, MI, USA). Lipid peroxidation was evaluated by the formation of MDA as the product of thiobarbituric acid-reactive substances (TBARSs). Samples, standards and all reagent enzymes were prepared and performed as described by the manufacturer’s instructions (TBARS Assay Kit; Cayman Chemical Co.). The values were expressed as μmol/L of MDA.

All assays were performed in duplicate using different dilutions of samples. Plates were read in a microplate spectrophotometer (Epoch; BioTek Instruments Inc.).

### Statistical analysis

Data were expressed as mean ± standard error of mean (SEM) of four to five experiments. Unpaired Student’s *t*-test and one-way analysis of variance (ANOVA) followed by Tukey’s posttest were performed as appropriate to determine differences between groups using the program GraphPad Prism 6.0 (GraphPad Software, La Jolla, CA, USA). A significance level of *p* <0.05 was adopted.

## Results

**Figure 1:**
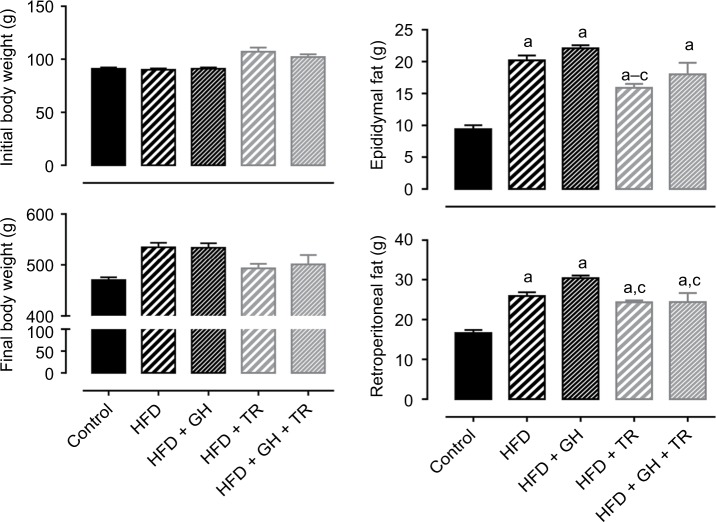
Body weight and fat accumulation. **Notes:** Initial and final body weight and epididymal and retroperitoneal fat of control rats and rats fed with HFD, supplemented (or not) with GH and submitted (or not) to TR. Data are presented as mean ± SEM of five animals. ^a^*P* < 0.05 compared to the control group. ^b^*P* < 0.05 compared to the HFD group. ^c^*P* < 0.05 compared to the HFD + GH group. **Abbreviations:** GH, glucosyl hesperidin; HFD, high-fat diet; SEM, standard error of the mean; TR, physical training.

In this study, we first evaluated the effects of antioxidant supplementation or physical training on the body weight of animals fed with HFD. No differences in food consumption were observed among all groups. The physical training group (TR) presented a higher intake of water which was not related to the beginning of the physical training, since the daily average of water intake after the fourth week of the study was 53 ± 4 mL/rat/day. Nonetheless, the total energy intake (kcal) was significantly higher in all animals fed with HFD, since fructose provided 0.4 kcal/mL and HFD provided 5.5 kcal/g compared to 3 kcal/g provided by the standard chow. Data are summarized in Table 2. Rats exhibited similar body weight at the beginning of the study. After 12 weeks of HFD, the body weight gain was higher when compared to animals fed with standard chow (control group). Treatment with GH only did not prevent body weight gain induced by HFD, while 8 weeks of physical training prevented exacerbated body weight gain (there was no significant increase when compared to the control group). Supplementation with GH did not cause any further effect in preventing body weight gain in exercised animals. VAT was estimated by weighing epididymal and retroperitoneal fat. Rats fed with HFD exhibited a higher accumulation of epididymal and retroperitoneal fat when compared to the control group. Epididymal fat was significantly less in rats subjected to physical training (HFD + TR) compared to HFD and HFD + GH. However, this decrease was not significant in animals receiving both GH supplementation and physical training. Retroperitoneal fat was higher in all groups fed with HFD compared to the control group. Trained animals receiving or not receiving GH presented decreased retroperitoneal fat when compared to sedentary HFD + GH (Figure 1).

**Figure 2:**
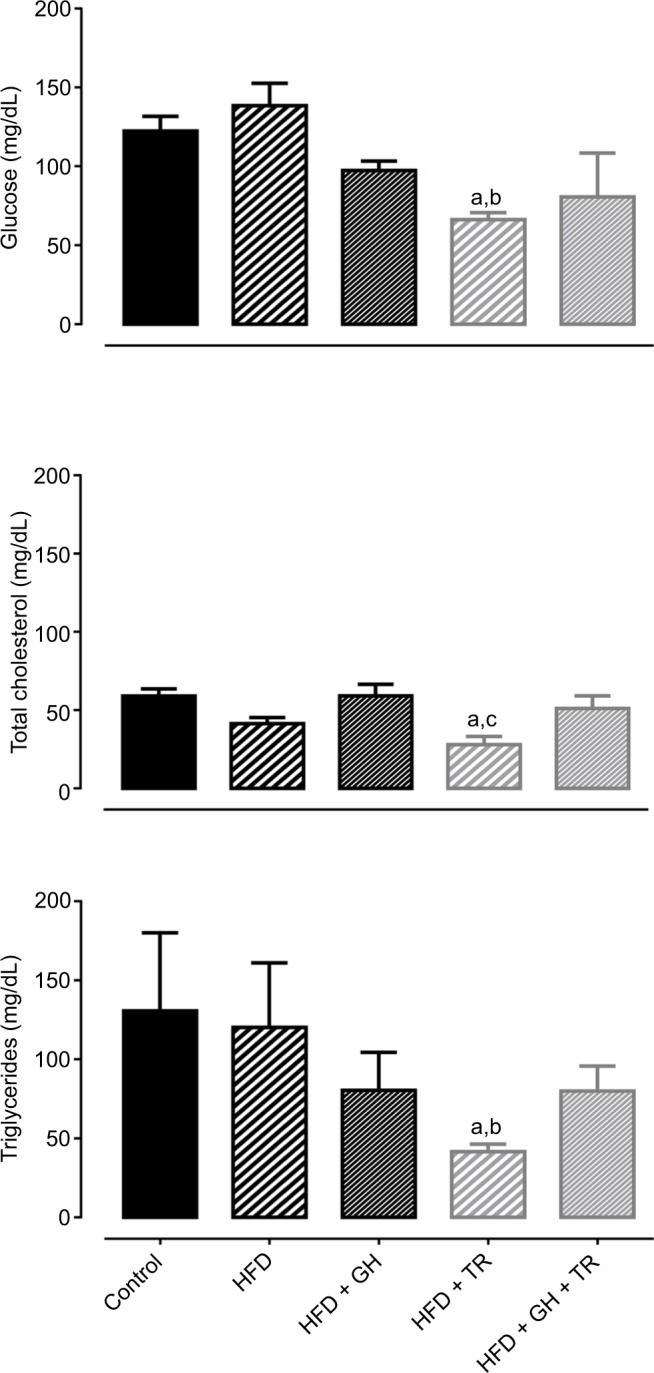
Plasma glucose and lipid levels. **Notes:** Plasma levels of glucose, total cholesterol and triglycerides of control rats and rats fed with HFD supplemented or not with GH and submitted or not to TR. Data are presented as mean ± SEM of four to five animals. ^a^*P* < 0.05 compared to the control group. ^b^*P* < 0.05 compared to the HFD group. ^c^*P* < 0.05 compared to the HFD + GH group. **Abbreviations:** GH, glucosyl hesperidin; HFD, high-fat diet; SEM, standard error of the mean; TR, physical training.

Consumption of HFD as well as supplementation with GH did not change plasma levels of fasting glucose. On the other hand, physical training significantly lowered glycemia below control levels. Glucose levels of sedentary rats tended to decrease following supplementation with GH (HFD + GH). Similarly, plasma levels of total cholesterol and triglycerides were not changed by supplementation with GH, whereas physical training significantly reduced plasma levels of total cholesterol and triglycerides compared to the control group. On the other hand, plasma lipid levels of HFD + GH + TR were not reduced (Figure 2).

**Figure 3:**
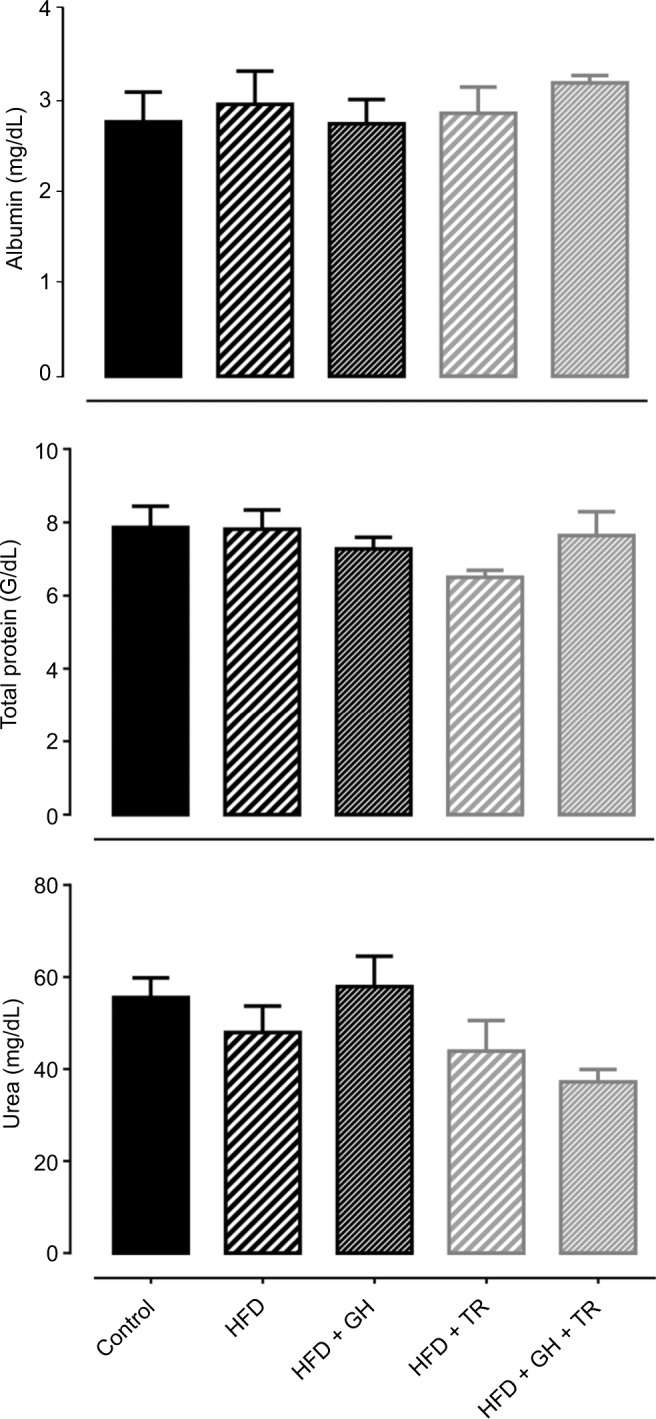
Hepatic and renal function. **Notes:** Serum levels of albumin, total proteins and urea from control and hyperlipidemic (HFD) rats, supplemented or not with GH and subjected to TR after 12 weeks of the study. Data are presented as mean ± SEM of four to five animals. **Abbreviations:** GH, glucosyl hesperidin; HFD, high-fat diet; SEM, standard error of the mean; TR, physical training.

Hepatotoxicity and nephrotoxicity of GH supplementation were evaluated by serum levels of albumin + total protein and urea, respectively. Serum levels of these metabolites remained unchanged in all the experimental groups (Figure 3).

**Figure 4:**
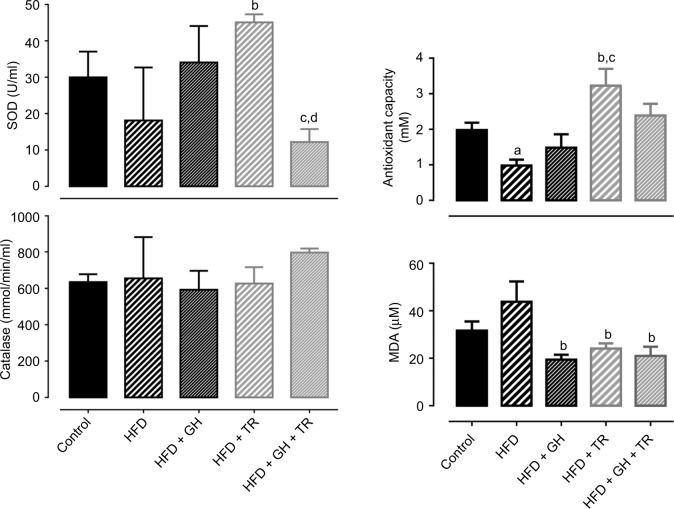
Oxidative status. **Notes:** Plasma activity of antioxidant enzymes SOD and CAT and TAC and MDA concentration (taken as lipid peroxidation index). Data are presented as mean ± SEM of three to five animals. ^a^*P* < 0.05 compared to the control group. ^b^*P* < 0.05 compared to the HFD group. ^c^*P* < 0.05 compared to the HFD + GH group. ^d^*P* < 0.05 compared to the HFD + TR group. **Abbreviations:** CAT, catalase; GH, glucosyl hesperidin; HFD, high-fat diet; MDA, malondialdehyde; SEM, standard error of the mean; SOD, superoxide dismutase; TAC, total antioxidant capacity; TR, physical training.

Effects of each treatment on the modulation of oxidative status were evaluated by the activity of endogenous antioxidant enzymes such as SOD and CAT as well as TAC. SOD activity was not changed by the HFD or GH group, while physical training increased SOD activity in plasma, although this increase was suppressed by supplementation with GH. CAT activity was not changed in any experimental group. In addition, TAC was decreased in the HFD group, which was reversed by physical training. GH improved TAC in the sedentary group but blunted the increase promoted by physical training. Lipid peroxidation was reduced in the HFD + GH and HFD + TR + GH groups, when compared to the HFD group. However, this effect was more pronounced in exercised rats which were not receiving GH as a supplement (GH + TR; Figure 4).

Endothelium-dependent relaxation was obtained by concentration–response curves to ACh in mesenteric artery rings with intact endothelium. Table 1 summarizes the potency (pharmacological parameter which is given by the negative logarithm of the effective concentration of the drug to produce 50% of the maximal response [pEC_50_]) and maximal response (E_max_) induced by ACh in each experimental group. HFD diminished the potency of ACh in this vascular bed. Neither GH supplementation nor physical training might restore the potency of ACh. Conversely, a worsened potency was observed for ACh in all treatments employed. E_max_ to ACh was reduced only in rats subjected to GH supplementation (Table 3).

**Figure 5:**
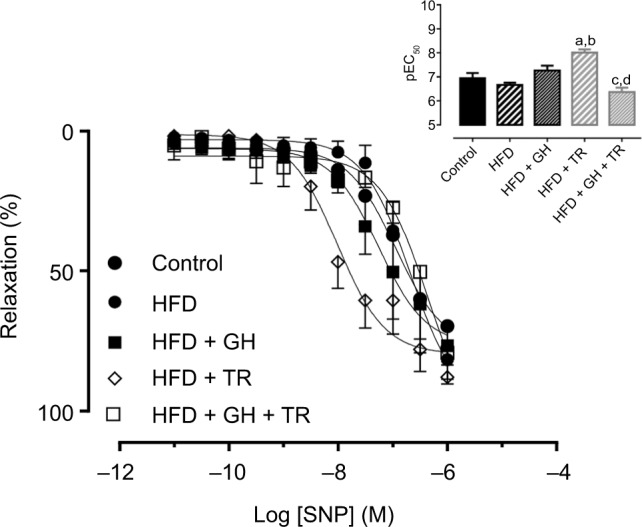
Endothelium-independent relaxation. **Notes:** Concentration–response curves to SNP in endothelium-denuded mesenteric artery of control rats or fed with HFD, supplemented with GH and/or subjected to TR. The inset shows the graph representation of the potency of SNP for each group. Data are presented as mean ± SEM of three to four animals. ^a^*P* < 0.05 compared to the control group. ^b^*P* < 0.05 compared to the HFD group. ^c^*P* < 0.05 compared to the HFD + GH group. ^d^*P* < 0.05 compared to the HFD + TR group. **Abbreviations:** GH, glucosyl hesperidin; HFD, high-fat diet; pEC_50_, potency (pharmacological parameter which is given by the negative logarithm of the effective concentration of the drug to produce 50% of the maximal response); SEM, standard error of the mean; SNP, sodium nitroprusside; TR, physical training.

Finally, endothelium-independent vasorelaxation was evaluated by concentration–response curves to NO donor SNP obtained in mesenteric artery rings with denuded endothelium. The potency of SNP was not changed in this vascular bed of rats fed with HFD (pEC_50_ = 6.67 ± 0.06) compared to the control group (pEC_50_ = 6.95 ± 0.21). Similarly, GH supplementation alone did not affect the potency of SNP (pEC_50_ = 7.26 ± 0.20). However, physical training significantly increased the potency of the SNP (pEC_50_ = 8.01 ± 0.13) when compared either to the control or HFD group. On the other hand, in exercised rats supplemented with GH, the potency of SNP was significantly reduced (pEC_50_ = 6.37 ± 0.18) compared to animals supplemented with GH only or subjected to physical training only (Figure 5).

## Discussion

In this study, we evaluated the effects of 8 weeks of supplementation with the antioxidant flavonoid, GH, associated (or not) with physical training on body weight and fat accumulation, biochemical parameters, oxidative status and vascular function of rats fed with HFD plus fructose. Rats fed with HFD presented a significant higher body weight compared to the control group, which was not prevented by GH supplementation. This is according to a previous study showing that mice made obese with an HFD did not exhibit lower weight gain when supplemented only with GH, although in association with caffeine (but not caffeine only) there was a decrease in the body weight gain.^23^ On the other hand, physical training prevented exacerbated body weight gain, keeping values similar to those observed in the control group. It is important to note that animals in this study were kept on HFD while the training program was being conducted. Endurance exercise is known to decrease the body weight gain in rats fed with HFD; however, better results are seen after HFD is replaced by a standard diet.^24^,^25^ No further effect on preventing body weight gain was observed with the association of GH supplementation, corroborating another study, where vitamin E was administered as an antioxidant in exercised obese rats.^26^ In addition, VAT was estimated by weighing epididymal and retroperitoneal fat. All experimental groups that fed with HFD exhibited higher fat depot than the control group. Nevertheless, the HFD + TR group showed a decrease in the epididymal fat pad compared to the HFD and HFD + GH groups, showing the positive effect of exercise in preventing fat accumulation. On the other hand, this effect was not seen when exercised animals were supplemented with GH, suggesting a partial inhibition of the effects of physical training. Antioxidant flavonoids are known to inhibit lipogenesis; therefore, an antiobesity effect would be expected. It was previously shown that flavonoids such as naringenin, rutin, hesperidin, resveratrol, naringin and quercetin were able to induce apoptosis in cell culture of pre-adipocytes with higher activity for quercetin.^27^ However, supplementation with antioxidant flavonoids fail to demonstrate antiobesity effects when associated with HFD.^28^,^29^ Similarly, studies show that, in association with physical exercise, antioxidant substances did not promote additional loss of weight.^26^,^30^ Eutrophic rats subjected to 12 weeks of swimming had a significant reduction in body weight gain after either continuous or interval training; however, GH concomitantly does not affect the body weight gain.^30^ It corroborates our finding that GH supplementation did not prevent body weight gain and fat accumulation in either sedentary or trained rats. Although trained animals exhibited diminished body weight gain, higher fat depot was observed compared to control animals, which suggests that trained animals may have lost part of lean body mass. It could be a consequence of the characteristics of HFD, which has reduced the percentage of carbohydrates, in combination with the energetic needs of physical exercise and recovery, which might increase the breakdown of proteins to generate glucose through gluconeogenesis.

As mentioned earlier, lipid metabolism is modulated by flavonoids, which might reduce plasma lipids and, hence, might be an important tool for controlling dyslipidemia. Therefore, in this study, we measured fasting plasma levels of glucose, total cholesterol and triglycerides. Our results showed that only physical training was able to decrease these parameters. We have previously demonstrated that, in healthy rats, treatment with hesperidin, GH or hesperetin did not change plasma lipids when compared to control animals.^14^ In another study, however, GH or continuous exercise itself reduced serum levels of glucose, total and low-density lipoprotein (LDL) cholesterol and triglycerides and increased high-density lipoprotein (HDL) cholesterol fraction in healthy animals, while the combination of GH and continuous exercise caused a further reduction only in total and LDL cholesterol.^30^ In a pathological condition, 6-week treatment with neohesperidin decreased fasting glycemia, insulin resistance and serum levels of cholesterol and triglycerides of diabetic mice.^31^ In rats fed with hypercholesterolemic diet, 12 weeks of hesperidin decreased plasma levels of cholesterol and reduced hepatic steatosis with no changes in serum levels of LDL cholesterol and triglycerides. In addition, higher levels of cholesterol were found in feces, suggesting that hesperidin decreases cholesterol absorption in the gastrointestinal tract and controls gene expression of proteins related to protein metabolism.^32^ In our study, we observed only a tendency of GH to modulate plasma levels of glucose (*P* = 0.1148) and triglycerides (*P* = 0.1638). It is possible that these controversial findings regarding the antiobesity and antilipemic activities of flavonoids are due to the type of flavonoid tested, experimental model differences, duration and dose employed in each study. In addition, we chose a model of HFD in an attempt to evaluate the effects of the physical exercise and/or antioxidant treatment in conditions of altered plasma glucose or lipids. However, our treatment with HFD failed in increasing fasting glucose and lipids. Our previous study showed that HFD + fructose for 12 weeks increased the accumulation of lipids in the liver. Thus, we speculate that HFD + fructose should be administered for a prolonged period to cause an increase in plasma levels of lipids. In fact, more studies are necessary to comprehend particularities of GH on the body weight composition and biochemical parameters and its effects when associated with physical exercise.

During absorption, flavonoids undergo several structural modifications to be absorbed and transported to the liver by the hepatic portal system. In the liver, flavonoids are biotransformed through Phase II conjugation enzymes, Uridine 5′-diphospho glucuronosyltransferases and sulfotransferases, forming several metabolites.^33^ Some of these metabolites may become more polar, and thus may be eliminated more easily from the body and could be detected in feces, bile or urine.^34^,^35^ Because flavonoids undergo liver and kidney metabolism, we evaluated hepatic and renal function of the experimental groups through the serum levels of albumin and total protein (for hepatic function) and urea (for renal function), as an index of hepatotoxicity and nephrotoxicity, respectively. No changes were observed in serum levels of these markers, indicating that GH has no hepatotoxic or nephrotoxic effect. It is in accordance with previous findings showing that hesperidin has a protective effect against drug-induced hepatotoxicity and nephrotoxicity.^36^–^38^

Flavonoids are known for their antioxidant activity; thus, we investigated the oxidative status by measuring plasma activity of endogenous antioxidant enzymes SOD and CAT as well as the TAC and lipid peroxidation. In all groups studied, CAT was not altered. However, SOD activity was significantly increased by physical training, although association with GH negatively reverted this effect, decreasing SOD activity compared to HFD + GH and HFD + TR. Physical training also improved the TAC. GH did not alter TAC in either sedentary or trained rats. On the other hand, lipid peroxidation was reduced by GH, physical training or both together. Studies have shown that physical training modulates SOD activity, increasing TAC and decreasing lipid peroxidation in experimental and human models.^39^,^40^ On the other hand, there is evidence that treatment with exogenous antioxidant agents suppresses the beneficial effects of physical exercise on the oxidative status. A previous study suggested that physical training causes a temporary increase in the production of ROS, which might stimulate the synthesis of a greater quantity of endogenous antioxidant enzymes. However, exogenous antioxidants could react with ROS, inhibiting the stimulus for the endogenous synthesis of antioxidant enzymes.^17^ In addition, it was recently demonstrated that the acute administration of an antioxidant cocktail impaired cycling performance and physiological function of young healthy males under normoxia or hyperoxia, suggesting that antioxidant consumption may compromise the balance between pro- and antioxidant systems.^41^ Together, these data show that exogenous antioxidant may not always play a positive role for redox balance, and it corroborates our findings since GH supplementation prevented some of the modifications induced by physical exercise on the oxidative status. However, literature shows that the antioxidant supplementation has been extensively studied and many controversial results have been described. The differences observed are probably related to the physiological or pathological conditions, to different antioxidant compounds and dose and route of administration, duration of the supplementation, etc. A previous study showed that rats fed with a cafeteria diet and supplemented with quercetin – another flavonoid – exhibited an improvement in redox state by increasing the activity of antioxidant enzymes such as CAT and reduced glutathione (GSH) and decreasing the formation of ROS (anion superoxide and nitric oxide) and lipid peroxidation (MDA formation and carbonylation of proteins).^42^ This is in agreement with our results showing that GH supplementation given alone reduced lipid peroxidation. It is suggestive that, in some pathological conditions, exogenous administration of antioxidants may be of interest to protect cells and organs against the increased production of ROS and impaired endogenous antioxidant defense.

Obesity may cause vascular disease, and hence, we evaluated endothelium-dependent and endothelium-independent relaxation in rat mesenteric arteries through concentration–response curves to ACh and SNP, respectively. A previous study showed that ACh-induced relaxation was impaired in aorta and mesenteric artery of rats fed with hypercaloric diet which was reversed by physical training.^43^,^44^ It is in accordance with our findings showing that the potency of ACh was reduced in the HFD group and also corroborates our previous data showing reduced potency of ACh in mesenteric artery of rats fed with HFD.^19^ However, we failed to demonstrate that physical training had a preventive effect on endothelial dysfunction induced by HFD. In hamsters fed with HFD and subjected to physical training, endothelial function improvement was more pronounced when associated with changes on diet.^45^ It might be suggestive that the prevention of cardiovascular disease is dependent on dietary modifications in addition to a physical training program. Thus, we speculate that adjustments in diet habits as well as duration and intensity of physical training program might be helpful to prevent the endothelial damage. In addition, GH supplementation reduced the potency of ACh in mesenteric arteries of either sedentary or trained rats. A previous study demonstrated that treatment with flavonoids (hesperidin, naringin and GH) plays a protective role in endothelial dysfunction in hypertensive rats.^46^–^48^ However, in rats supplemented with L-carnitine, which has antioxidant properties, we have found impaired endothelium-dependent relaxation in mesenteric artery of sedentary and trained lean rats, corroborating our data that exogenous antioxidants may occasionally impair vasodilation.^49^,^50^ It is well known that several antioxidants derived from plants and fruits improve endothelial and vascular function by decreasing oxidative stress.^51^ However, since NO is a free radical, antioxidants may also scavenge NO. In vitro, recent studies showed that the extract of Chilauni (which is an antioxidant with high content of flavonoids) and cranberry flavonoids were able to scavenge NO and other free radicals as well.^52^–^54^ Therefore, it is possible that exogenous antioxidants scavenged NO released from endothelial cells, diminishing the potency of ACh in animals supplemented with GH. Further, vascular function was assessed also by endothelium-independent relaxation induced by the NO donor SNP in mesenteric rings with denuded endothelium, which was not affected by HFD or GH. It was previously shown that the relaxation induced by SNP is not altered by diet or physical training.^43^–^45^ Nevertheless, in this study, physical training increased the potency of SNP, although this effect was inhibited when GH was administered concomitantly, suggesting that exogenous antioxidants in fact impair the benefits promoted by physical exercise not only on the oxidative status but also in the vascular reactivity.

Taken together, these data are suggestive that antioxidant compounds might be useful in pathological conditions where there is an increase in the production of oxidative substances. However, supplementation with antioxidants might be avoided under normal physiological conditions or associated with TR, since exogenous antioxidants inhibit organism adaptations elicited by endogenous stimuli in healthy conditions or it blunts the effects of physical exercise.

Our study, however, has some limitations. These results reflect a specific combination of low-intensity aerobic exercise and the antioxidant flavonoid, GH, in a model of obesity induced by HFD. Furthermore, besides the fact that we performed experiments only in male rats, it is also necessary to be careful in extrapolating the results to humans, considering human metabolism and differences in the environment. Although many in vitro strategies might be used to analyze the effects of GH in human cells, the association with physical exercise would be limited.

## Conclusion

Our findings demonstrate that, in an experimental model of obesity, GH supplementation improved TAC and decreased lipid peroxidation in sedentary rats fed with HFD. Physical training decreased body weight, fat depot and plasma levels of glucose and lipids and improved the oxidative status and endothelium-independent relaxation of the mesenteric artery. Nevertheless, GH given in association with physical exercise did not cause further changes in addition to those promoted by physical exercise. On the contrary, in exercised animals, GH prevented those changes elicited by physical training in plasma glucose and lipid levels, oxidative status and endothelium-independent relaxation.

## Figures and Tables

**Table 1:** Dietary composition of high-fat diet compared to standard chow

Dietary composition			
Standard chow (g/kg)		High-fat diet (g/kg)	
Cornstarch	465.7	Cornstarch	19.5
Maltodextrin	155	Dextrinated starch	150
Casein	140	Casein	200
Sucrose	100	Sucrose	150
Cellulose fibers	50	Cellulose fibers	50
Soy oil	40	Soy oil	30
Mineral mix	35	Mineral mix	35
Vitamin mix	10	Vitamin mix	10
L-cystine	1.8	L-cystine	3
Choline	2.5	Choline	2.5
TBHQ (tert-butyl hydroquinone)	0.008	BHT (butylated hydroxytoluene)	0.014
Lard	0	Lard	350

**Note:** High-fat diet composition data taken from the manufacturer’s 2016 data-sheet (Prag Solutions Biosciences, Jau, Brazil).^55^

**Table 2:** Daily average of water, food and energy intake

Parameter/group	Control	HFD	HFD + GH	HFD + TR	HFD + TR + GH
Water intake (mL/rat/day)	39 ± 3	42 ± 1.5	42 ± 2	48 ± 2^a^,^b^	40 ± 1
Food consumption (g/rat/day)	13 ± 1	12 ± 0.3	12 ± 0.4	11 ± 0.5	11 ± 0.3
Total energy (kcal/rat/day)	38 ± 1	81 ± 2^a^	83 ± 3^a^	82 ± 3^a^	78 ± 2^a^

**Notes:** Data are presented as mean ± SEM for 71–74 days.

a *P* < 0.05 compared to the control group.

b *P* < 0.05 compared to the HFD + TR + GH group.

**Abbreviations:** GH, glucosyl hesperidin; HFD, high-fat diet; SEM, standard error of the mean; TR, physical training.

**Table 3:** pEC_50_ and E_MAX_ of ACh in mesenteric artery with intact endothelium of control rats and rats fed with HFD, submitted (or not) to GH supplementation and/or submitted (or not) to physical exercise (TR)

ACh	Group	Control	HFD	GH
pEC_50_	Sedentary or non-TR	7.36 ± 0.08	6.83 ± 0.21^a^	6.25 ± 0.14^a^,^b^
	TR	Not applicable	6.17 ± 0.10^a^,^b^	6.30 ± 0.21^a^,^b^
E_max_	Sedentary or non-TR	96 ± 0.3	92 ± 2	70 ± 15^a^,^b^
	TR	Not applicable	88 ± 7	92 ± 3

**Notes:** Data are presented as mean ± SEM for three to four animals.

a *P* < 0.05 compared to the control group.

b *P* < 0.05 compared to the HFD group.

**Abbreviations:** ACh, acetylcholine; E_max_, maximal response; GH, glucosyl hesperidin; HFD, high-fat diet; pEC_50_, potency (pharmacological parameter which is given by the negative logarithm of the effective concentration of the drug to produce 50% of the maximal response); SEM, standard error of mean; TR, physical training.
